# Dr. L. Ch. Boisliniere

**Published:** 1896-02

**Authors:** 


### Obituary.

Dr. L. Ch. Boisliniere, of St. Louis, died at his home, 3509 Olive street, in that city, January 13, 1896, aged 80 years.

He was born (*Weekly Review*), in 1816 on the Island of Guadaloupe, in the West Indies. At an early age he went to France, and thinking first to make the law his profession, he graduated in law at the University of Paris. He soon began the study of medicine, however, and his interest in the science was so marked that he was made a member of the Anthropological Society of Paris, and under its auspices spent several months in traveling in South America. He maintained his connection with the Society until his death.

When he returned to this country, in 1846, he brought letters of introduction to Henry Clay, and for some time he made his home in Louisville, Ky. He continued his medical studies and graduated at the St. Louis Medical College in 1848.

In 1870 he was appointed professor of obstetrics at the St. Louis medical college, succeeding Dr. M. M. Pallen.

The degree of doctor of laws was conferred upon him by the same institution in 1879, and at his death he held the position of emeritus professor of obstetrics. He was a frequent contributor to medical journals, and a short time ago he completed a work entitled obstetric accidents, emergencies and operations, which has been adopted as a text-book in a number of medical colleges.

He enjoyed, in a high degree, the respect and admiration of his colleagues, patients and friends, and was universally liked, both socially and professionally.

Dr. Boislinière was a member of the St. Louis medical society, of the gynecological and obstetrical society, and an honorary Fellow of the American association of obstetricians and gynecologists. He leaves a widow, five daughters and a son, the latter a well-known physician in St. Louis.

